# Nilotinib-Induced Diabetes Mellitus in a Young Female with Chronic Myeloid Leukemia

**DOI:** 10.7759/cureus.10277

**Published:** 2020-09-06

**Authors:** Abdel-Latif S Ismail, Mohamed A Yassin

**Affiliations:** 1 Internal Medicine Department, Hamad Medical Corporation, Doha, QAT

**Keywords:** hyperglycemia, tyrosine kinase inhibitors, insulin resistance, cml, chronic myelocytic leukemia, diabetes mellitus

## Abstract

Chronic myeloid leukemia (CML) is one of the myeloproliferative neoplasms whose incidence peaks in older individuals and is characterized by the uncontrolled proliferation of cells in the myeloid cell line. It can present with symptoms related to leukocytosis or splenomegaly, and it can also be asymptomatic and found incidentally. Nilotinib is a second-generation tyrosine kinase inhibitor used in the treatment of the chronic phase of CML and was found to cause diabetes mellitus among other adverse effects. These adverse effects seem to occur after a few months to a few years of drug administration. Type-two diabetes is the most common type of diabetes; its hallmark being insulin resistance with resultant hyperglycemia. Most often, it has no symptoms and is usually found as an incidental finding or on screening. Here we present a 45-year old Chinese female, who was diagnosed with CML chronic phase after an incidental finding of leukocytosis; treated with nilotinib as upfront for CML, and developed diabetes mellitus after about one and a half years of treatment, which was treated with metformin.

## Introduction

Chronic myeloid leukemia (CML) is a myeloproliferative neoplasm characterized by the dysregulated production and uncontrolled proliferation of mature and maturing granulocytes with fairly normal differentiation. It mainly affects adults and is rarely seen in children. The average age at diagnosis is around 64 years [1]. CML typically runs a biphasic or triphasic course (chronic phase, accelerated phase, and blastic phase), with a diagnosis usually made in the chronic phase. Without effective treatment, patients eventually progress to a blastic phase, frequently through an intermediate or accelerated phase [2].

Generally, CML is one of the best examples of effective targeted therapy. Tyrosine kinase inhibitors have transformed the outcomes of patients with CML over the last 20 years. Today, the overall survival of patients with CML treated by tyrosine kinase inhibitors (TKIs) is very close to that of the healthy population [3]. TKIs act by interfering with the interaction between the BCR-ABL oncoprotein and adenosine triphosphate, thereby blocking the proliferation of the malignant clone. There are currently three TKIs that are approved by the Food and Drug Administration (FDA) for the first-line treatment of chronic phase CML: imatinib, dasatinib, and nilotinib [4].

Among the known adverse effects related to these TKIs, nilotinib is known to lead to the development of diabetes mellitus; as well as cardiovascular complications, including hypertension [5]. Our case report here is about a middle-aged Chinese female who was not known to have chronic diseases and who was diagnosed with CML after an incidental finding of leukocytosis. She was treated with nilotinib upfront for chronic phase CML, with a good molecular response; however, she developed diabetes mellitus after about one and a half years of treatment, and she was treated with metformin.

## Case presentation

This is a 45-year-old female, previously healthy, who was referred to the National Center for Cancer Care and Research in February 2017 for further evaluation after her complete blood count (CBC) testing showed an incidental finding of leukocytosis with basophilia.

She denied having any symptoms at that time; and her physical examination was unremarkable apart from a palpable liver edge one finger below the costal margin and a palpable spleen edge three fingers below the costal margin. Body mass index (BMI) was within normal limits.

Complete blood testing showed a leukocyte count of 240,000 X 10^3/uL (normal value: 4 to 10 X 10^3/uL) with a left shift and basophilia. Other blood tests were within normal limits.

**Figure 1:**
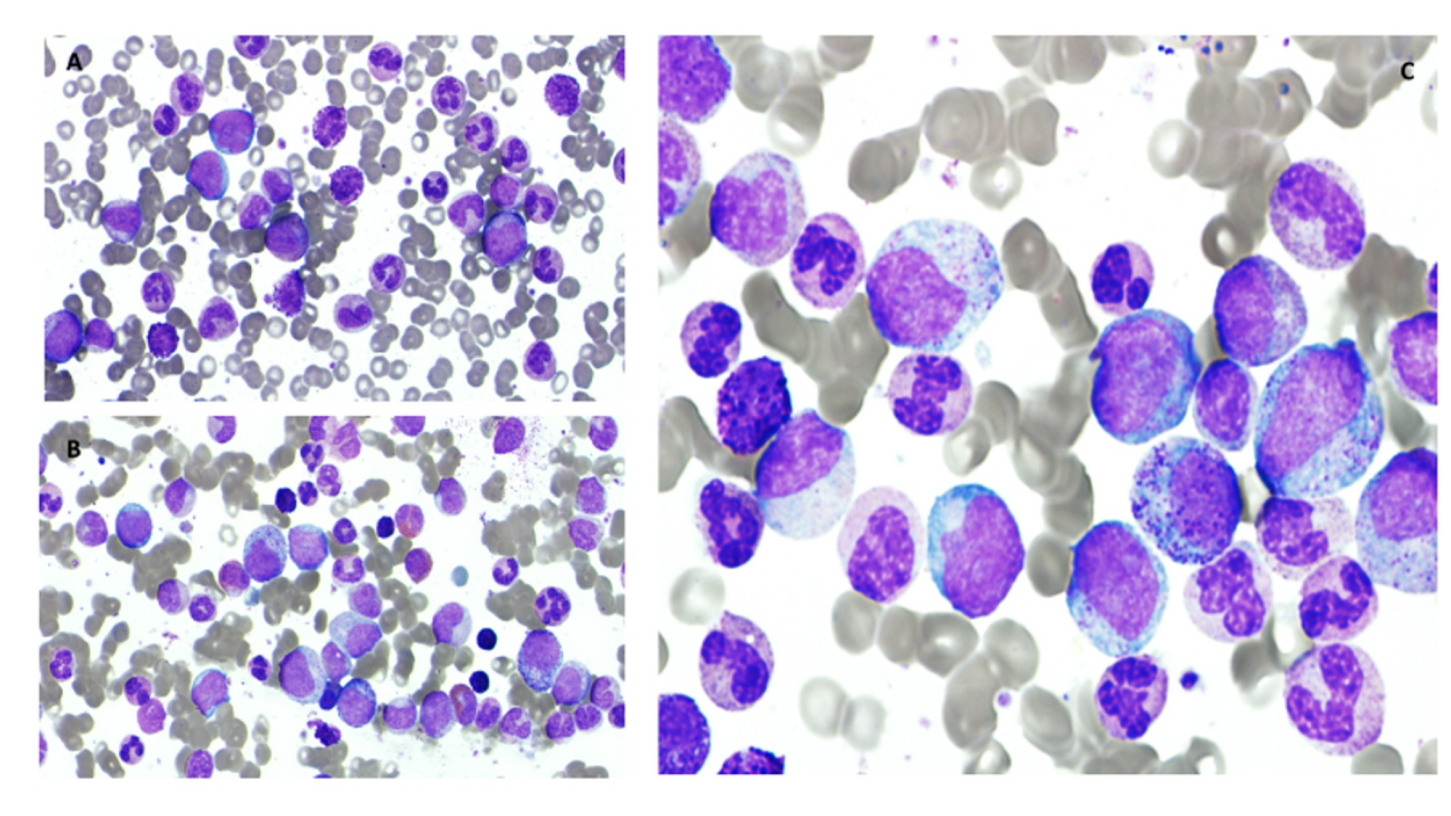
A: Peripheral blood smear, B and C: Bone marrow aspirate smear A. Peripheral blood smear showing hyperleukocytosis and neutrophilic cells at various stages of maturation, basophilia, and 1% blasts. (Wright Giemsa stain X500). B and C: Bone marrow aspirate smear is hypercellular with granulocytic hyperplasia, various stages of maturation with prominent basophils/eosinophils (X500) and ( X1000).

Peripheral blood smear showed hyperleukocytosis and neutrophilic cells at various stages of maturation, basophilia, and 1% blasts as shown in Figure 1A (Wright Giemsa stain X500). Bone marrow sampling was arranged, and bone marrow aspirate smear showed hypercellularity with granulocytic hyperplasia, various stages of maturation with prominent basophils/eosinophils as shown in Figure 1B (X500) and Figure 1C (X1000).

**Figure 2:**
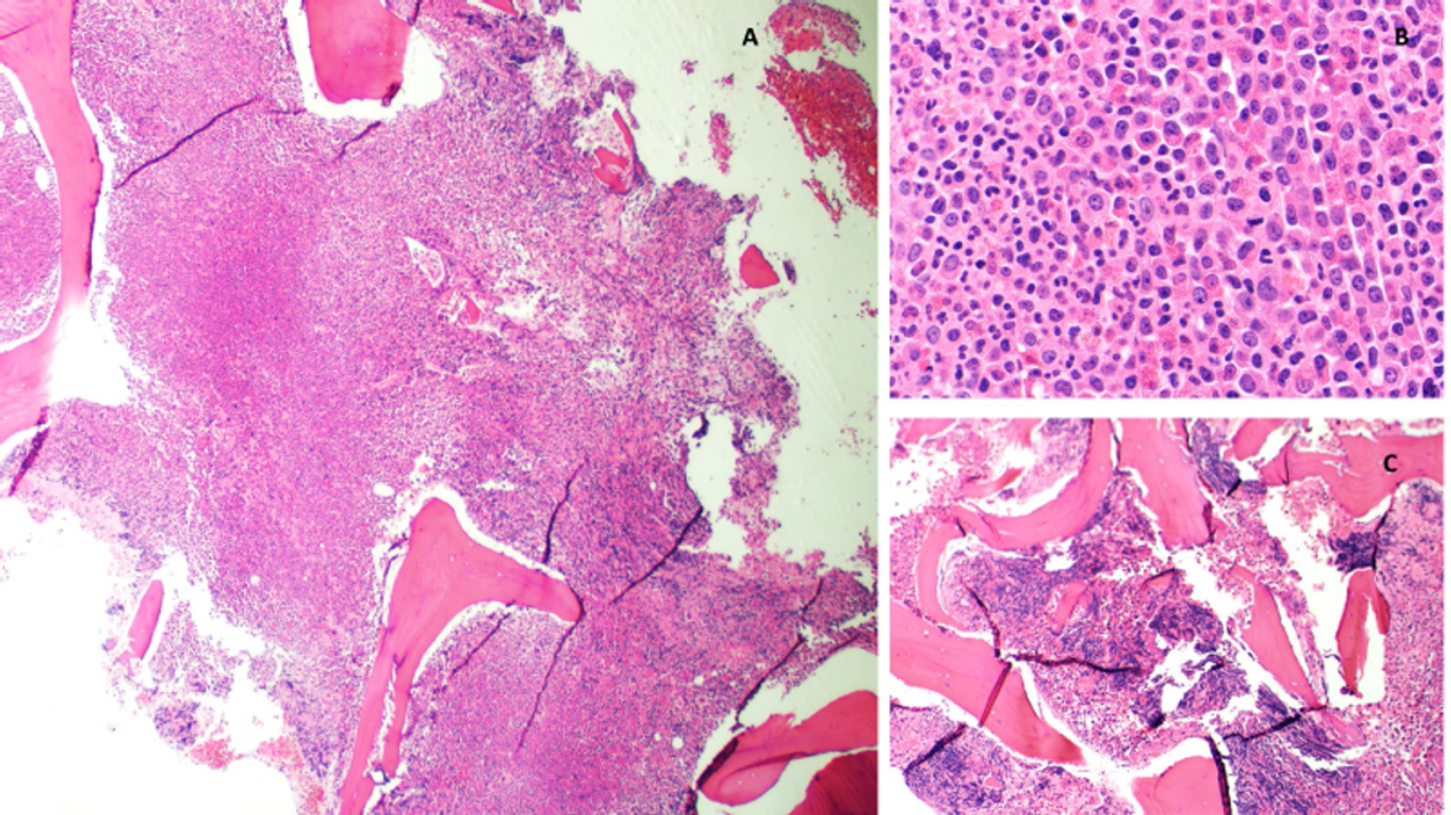
Bone marrow core biopsy A (X100), B (X500): Bone marrow core biopsy is extremely hypercellular with almost 100% cellularity, with marked granulocytic proliferation and prominent eosinophils. C: Focal areas of increased marrow fibrosis (MF1/3) with increased small/dwarf megakaryocytes (X100).

Bone marrow biopsy showed almost 100% cellularity with trilineage hematopoiesis and marked granulocytic proliferation as shown in Figure 2.

**Figure 3:**
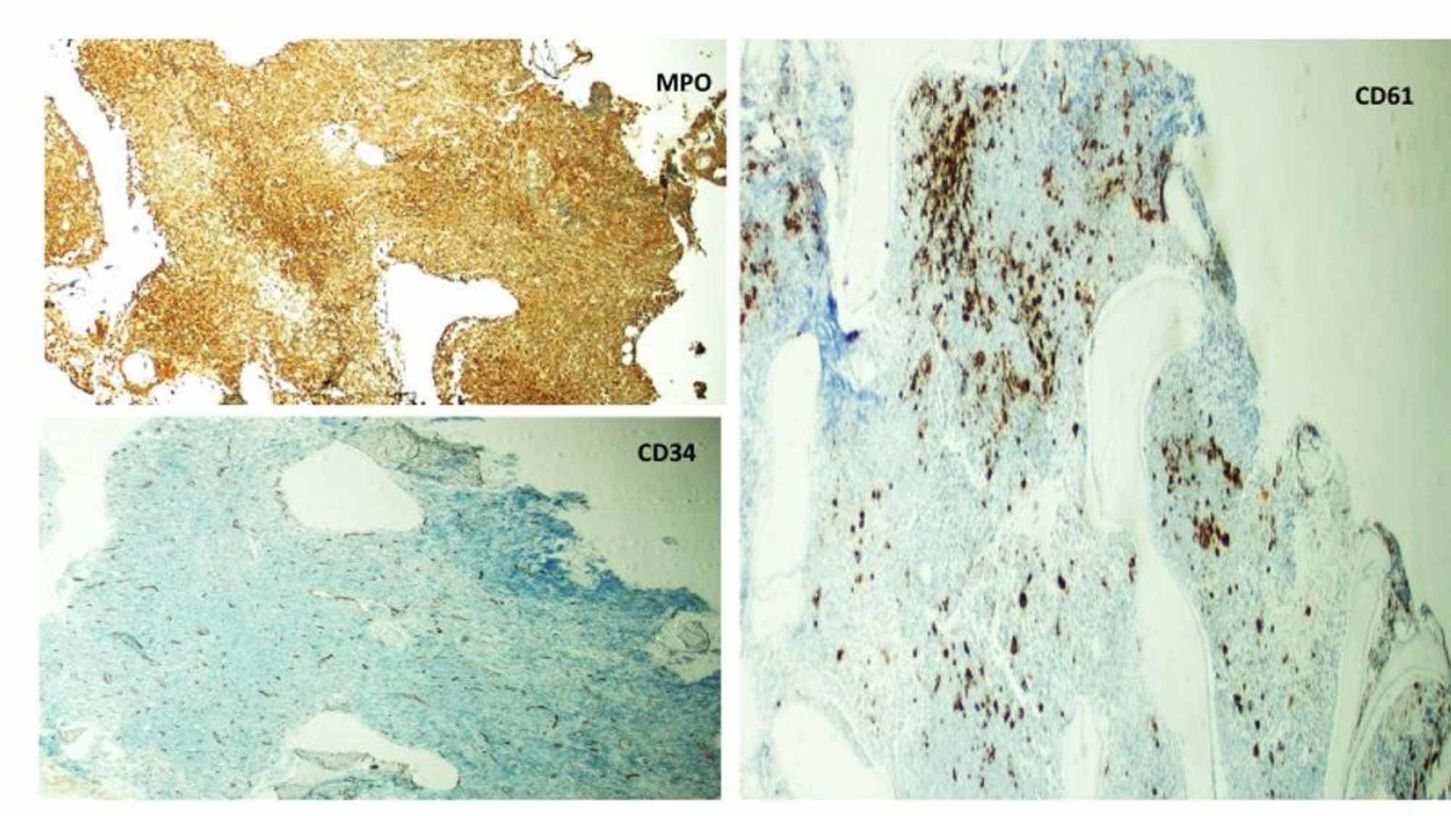
Bone marrow core biopsy immunohistochemical stains Bone marrow core biopsy immunohistochemical stains: CD34 showing no increase in blasts with increased marrow vasculatures; MPO highlighting the marked granulocytic hyperplasia; and CD61 highlighting the increased megakaryocytes with many dwarf forms.

Bone marrow core biopsy immunohistochemical staining was performed as shown in Figure 3. CD34 shows no increase in blasts with increased marrow vasculature; MPO highlights the marked granulocytic hyperplasia; and CD61 highlights the increased megakaryocytes with many dwarf forms.

Fluorescence in situ hybridization (FISH) analysis revealed an abnormal hybridization signal pattern with dual fusion indicating BCR/ABL1 rearrangement in 197/200 cells of the cells analyzed. These findings were consistent with chronic myelogenous leukemia in the chronic phase.

The patient was started on nilotinib upfront in March 2017, and follow up in the next months showed good response to treatment. Complete hematologic response (CHR), complete cytogenetic response (CCYR), and major molecular response (MMR) were achieved as per European LeukemiaNet (ELN) recommendations 2013 [6].

Eighteen months through her nilotinib regimen; specifically in August 2018, during one of her visits to the emergency department, her blood sugar was incidentally found to be high at 20 mmol/L. She didn’t have any specific symptoms of diabetes at that time. There was no family history of diabetes mellitus.

The patient was started on metformin and referred to the diabetes clinic; and the nilotinib regimen was continued without modification.

Glycated hemoglobin (HbA1c) was not done, and blood sugar readings in the following visits were within normal limits.

## Discussion

Chronic myeloid leukemia (CML, chronic myelocytic or chronic myelogenous leukemia) is associated with the Philadelphia chromosome t(9;22)(q34;q11) resulting in a BCR-ABL1 fusion gene. This genetic abnormality results in the formation of a unique gene product (BCR-ABL1), which is a constitutively active tyrosine kinase. This deregulated tyrosine kinase is implicated in the development of CML and has become a primary target for the treatment of this disorder [7]. Most patients present in chronic phase CML, which is typically manifest as leukocytosis with immature myeloid cells, with or without anemia, thrombocytopenia, constitutional symptoms, splenomegaly, and/or bleeding. Chronic phase CML can progress from a relatively indolent disorder to the more aggressive disorders, accelerated phase (AP) or blast crisis (BC) [1].

Available treatment options for patients with CML include allogeneic hematopoietic cell transplantation (HCT), tyrosine kinase inhibitors (TKIs), and palliative therapy with cytotoxic agents [1]. Oral TKIs target the constitutively active tyrosine kinase implicated in the pathogenesis of CML. These agents are able to achieve long-term control of CML in the majority of patients; thus, they are the initial treatment of choice for almost all newly diagnosed patients with CML [8]. All TKIs are associated with certain common adverse effects such as cytopenias, rash [9], nausea, edema, diarrhea [10], musculoskeletal [11], and fatigue early in the course of treatment.

These adverse effects typically arise in the first year of treatment, although some (e.g., cardiovascular adverse effects with dasatinib or nilotinib) may not emerge for many months or years. They are also mostly mild to moderate in intensity (ie, grade ≤2) and resolve spontaneously or can be controlled by dose adjustments.

Each TKI is also associated with particular adverse effects, some of which may not be manifest for months or years. Nilotinib is a second-generation TKI that has been associated with lower rates of progression to AP or BC than imatinib. It is associated with the following adverse effects: cytopenias, hepatotoxicity, QTc prolongation, pancreatitis, and long-term cardiovascular complications, as well as the development of diabetes mellitus, however, weight gain is not among the known adverse effects of nilotinib.

There are two proposed mechanisms for the development of nilotinib-induced diabetes:

I) Development of insulin resistance (including the possible role of nilotinib-induced adipokine alterations) II) Impaired insulin secretion (including the possible role of a nilotinib-induced perturbation of incretin secretion) [12]

We found two published studies that looked into that mechanism:

The first concluded that the mechanism for nilotinib-induced diabetes is post-receptor insulin resistance with the subsequent development of hyperinsulinemia [12]. The second concluded that nilotinib exacerbates diabetes mellitus by decreasing the secretion of endogenous insulin although the precise mechanism behind that is yet to be discovered [13].

Monitoring toxicity of all tyrosine kinase inhibitors (TKI) includes obtaining history, physical examination, laboratory studies, and specialized evaluations.

For nilotinib, physical examination should focus on the presence of jaundice, hepatomegaly, abdominal tenderness that may suggest liver disease or pancreatitis, pallor or reduced pulses of extremities, carotid bruit, cardiac dysrhythmia, and edema that may suggest cardiovascular disease.

Strategies to ameliorate adverse effects include symptomatic management, avoidance of medications that may exacerbate the toxicity, and judicious TKI dose adjustment or brief interruption. However, dose reductions should be undertaken with careful monitoring of the molecular response to avoid administering a sub-therapeutic dose. Extended interruptions of TKI treatment should be avoided because they may affect disease outcomes.

## Conclusions

To the best of our knowledge, the management of nilotinib-induced diabetes doesn’t differ from that of type-two diabetes, and this adverse effect seems to respond to the same first-line agents used to manage type-two diabetes. Moreover, and since nilotinib can cause other cardiovascular effects, such as hypertension, hyperlipidemia, and atherosclerosis, a holistic approach towards cardiovascular risk factor reduction should be followed, including advice for smoking cessation, weight loss, and adequate aerobic exercise.
